# Association with Uncontrolled Hypertension in Thoracic Aortic Aneurysm Patients Referred to a Tertiary-Care Center

**DOI:** 10.3390/healthcare14040515

**Published:** 2026-02-18

**Authors:** Laura Ramlawi, Serge Sicouri, Rhian Touyz, Dimitrios E. Magouliotis, Francesco Cabrucci, Colleen Innes, Massimo Baudo

**Affiliations:** 1 Department of Cardiac Surgery Research, Lankenau Institute for Medical Research, Main Line Health, Wynnewood, PA 19096, USA; laura.ramlawi@gmail.com (L.R.); magouliotisd@mlhs.org (D.E.M.); francesco.cabrucci.6@gmail.com (F.C.); innesc@mlhs.org (C.I.); massimo.baudo@icloud.com (M.B.); 2 Marianopolis College, Montreal, QC H3Y 1X9, Canada; 3 McGill University Health Centre Research Institute, McGill University, Montreal, QC H4A 3J1, Canada; rhian.touyz@mcgill.ca

**Keywords:** hypertension, thoracic aortic aneurysm, uncontrolled, demographic, blood pressure

## Abstract

**Highlights:**

**What are the main findings?**

- Among patients with thoracic aortic aneurysm, 54.2% had uncontrolled hypertension.
- Areas with a higher rate of college graduates may be associated to lower rates of uncontrolled hypertension.

**What are the implications of the main findings?**

- TAA patients may not be receiving adequate BP control at the primary-care level.
- This could stem from poor patient compliance or from an ineffective BP medication regimen.

**Abstract:**

**Background**: International guidelines recommend tight blood pressure (BP) control in patients with thoracic aortic aneurysm (TAA). Hypertension in TAA patients has been associated with an increased rate of aneurysm growth and also with aortic dissection or aortic rupture. We aimed to study BP control in TAA patients referred by a primary or cardiology provider to a tertiary aortic management program. **Methods**: This retrospective study analyzed 3525 consecutive patients with confirmed TAA diagnosis referred by a primary-care or cardiology provider for management at the Lankenau Aortic Surgical Program between January 2021 and December 2024. Blood pressure was registered using an appropriately sized cuff and a calibrated automated sphygmomanometer. Clinical and demographic data were compared between patients with different stages of hypertension, based on the 2023 ESH guidelines. **Results**: The overall rate of above-target BP in TAA patients was 54.2% (1911/3525). From the hypertension group, Stage 1 (BP > 140/90) accounted for 53.4% (1020/1911) of patients, with Stage 2 (BP > 160/100) accounting for 12.6% (241/1911) and Stage 3 (BP > 180/110) for 1.8% (35/1911). Among associations of hypertension with demographic data by zip code, no significant differences were observed between groups with respect to race, median household income, or house value. There was a tendency of lower BP in patients from residential areas with higher rates of college graduates compared to those without college education (OR: 0.995; *p* = 0.059). **Conclusions**: Hypertension remains both highly prevalent and inadequately controlled in patients with TAA, even within specialized care environments. These findings emphasize the need for a more comprehensive approach to risk factor management to improve outcomes in this high-risk population.

## 1. Introduction

Thoracic aortic aneurysms (TAAs) are life-threatening vascular conditions characterized by a localized dilation of the thoracic portion of the aorta due to weakening of the arterial wall [1,2,3]. The most serious complications include aortic rupture and dissection, both potentially fatal if not treated emergently. A key challenge with TAAs is their often silent progression [4,5]. Many cases are asymptomatic and are discovered incidentally through imaging performed for unrelated conditions [4]. Consequently, preventive strategies and careful management of risk factors are essential to mitigate the risk of aneurysm dilatation and sudden cardiac events [2,3,6,7,8]. Among these risk factors, elevated blood pressure (BP) increases wall stress on the aneurysmal segment, accelerating its growth and making it more prone to rupture or dissection [5,6]. Accordingly, international hypertension guidelines recommend maintaining BP below 130/80 mmHg in patients with TAA [2,3,9]. Nevertheless, BP control in these patients may be suboptimal. Overall, uncontrolled hypertension currently remains a healthcare issue beyond patients with TAA [10,11]. Although treatment strategies have improved over time, hypertension control remains inadequate across the U.S. population overall [12].

Any specific evaluation of BP control in patients with an established diagnosis of TAA is currently limited, as most published studies have focused mainly on abdominal aneurysm [13]. Moreover, most studies examining the relationship between uncontrolled hypertension and TAA have primarily assessed the incidence or prevalence of aneurysmal disease among hypertensive populations [14,15,16]. While these studies have identified hypertension as a major risk factor for aneurysm development and progression, they provide limited insight into BP control following diagnosis of TAA. This represents a critical knowledge gap, as such studies provide only limited insight into the adequacy and determinants of BP control in this context. Specifically, the existing literature largely addresses hypertension as an etiological and prognostic factor for aneurysm formation, rather than evaluating whether BP targets are achieved in clinical practice once TAA is diagnosed, how frequently BP remains uncontrolled, and which patient- or care-related factors may influence post-diagnosis BP management. This distinction is clinically relevant, as BP control represents a potentially modifiable factor aimed at slowing aneurysm progression and reducing adverse aortic events after diagnosis. Therefore, the present retrospective study was designed to address this unmet need in known TAA patients referred to a tertiary-care aortic management program. This is the perfect setting for quantifying the degree of BP control in patients such as these. Blood pressure was recorded at surgical outpatient presentation following prior management by primary-care providers or cardiologists, thereby assessing whether BP control was already achieved in routine clinical practice after diagnosis, irrespective of surgical indication. The study also aimed to classify the patients’ hypertension stage and to examine the potential role of demographic factors related to BP control.

## 2. Materials and Methods

This retrospective study included 3525 consecutive patients with confirmed thoracic aortic aneurysm (TAA) referred to a surgical outpatient visit at the Lankenau Aortic Surgical Program, Lankenau Heart Institute, Main Line Health, Wynnewood, PA, USA, between January 2021 and December 2024. Referrals were made either by primary-care providers or by cardiologists as a first surgical visit. No exclusion criteria were applied. All patients had a confirmed diagnosis of TAA through prior imaging, which was verified upon presentation to the center. In this surgical context, the study aimed to evaluate BP control after prior consultations with other healthcare providers; therefore, all referred patients were included regardless of their surgical indication. Given the elevated risk of complications in this population, including aortic dissections and ruptures, adequate BP control should already be established at the time of referral.

Data supporting the findings of this study are available from the corresponding author upon reasonable request, subject to institutional approval. The study was conducted and reported in accordance with the STROBE (Strengthening the Reporting of Observational Studies in Epidemiology) guidelines for observational research (Table S1) [17].

During this first surgical evaluation, a trained nurse practitioner measured the patient’s systolic and diastolic BP at least twice (once per arm), and the mean of these values was reported. Blood pressure was registered in the seated position after at least 5 min of rest, using an appropriately sized cuff and a calibrated automated sphygmomanometer. Hypertension stages were defined according to the 2023 European Society of Hypertension (ESH) guidelines [18]. Classifications for hypertension severity begin at Stage 1 (≥140/90 mmHg), progressing to Stage 2 (≥160/100 mmHg), and Stage 3 (also called “hypertensive crisis”, ≥180/110 mmHg). Patients with TAA should aim for a BP < 130/80 mmHg. Patients were considered to have TAA if any section of their thoracic aorta was greater than or equal to 4.0 cm [2].

Only the basic demographic data, including age, sex, and body mass index (BMI), which was collected during the visit could be retrieved. Data were obtained by retrospectively querying the patient’s visits database. Socioeconomic indicators at the zip code level, including the percentage of white-race residents, the neighborhood education level (at least college degree education), median household income, and housing values, were derived from publicly available databases, including the U.S. Census Bureau, the United States Postal Service (USPS), and the Internal Revenue Service (IRS) [https://www.unitedstateszipcodes.org/, accessed on 4 August 2025]. No individual-level socioeconomic indicator was available.

### 2.1. Statistical Analysis

Categorical variables were reported as frequency and percentages, and were compared using the Chi-squared test. Normality of continuous variables was assessed using the Kolmogorov–Smirnov test. Variables with normal distribution were compared using Student’s t-test and presented as mean and standard deviation, while Wilcoxon’s rank-sum test was used for non-normally distributed data that were presented as median and interquartile range.

To evaluate the relationship between demographic variables and the presence of hypertension, univariable logistic regression analyses were conducted and reported as an odds ratio (OR) with 95% confidence interval (95% CI). Multivariable analysis included all variables with a univariable *p* value < 0.10. As regression analysis measures associations, there was no intention to evaluate causality. A heatmap illustrating the rate of uncontrolled hypertension by zip code was constructed to visualize geographic distribution. Only zip codes with at least 20 patients were included in the analysis.

A *p*-value < 0.05 was considered statistically significant. Data processing and analysis were performed using R software (version 4.4.2) via the RStudio environment (R Project for Statistical Computing, Vienna, Austria).

### 2.2. Patient and Public Partnership

Patients were not directly involved in the design, conduct, or analysis of this retrospective study. The research question was informed by the clinical priority of optimizing BP control in individuals with TAA, a key modifiable risk factor identified through routine outpatient care and a widely recognized patient management need. Because the study used existing clinical data obtained during standard visits, patients were not asked to participate in study design, recruitment, assessment of intervention burden, or selection of outcomes. Results will be shared with the broader patient community through presentations and educational materials within our vascular and cardiology outpatient clinics, emphasizing practical guidance for hypertension management in this population.

## 3. Results

The median age of the population was 74 years [IQR: 66; 82] in the hypertensive group and 74 years [IQR: 65.3; 81] in the non-hypertensive group, *p* = 0.116. Median BMI was significantly higher in hypertensive patients, at 28.9 [IQR: 25.40; 32.8], compared to patients without hypertension, median BMI in this latter group being 27.6 [IQR: 24.3; 31.3], *p* < 0.001. Most patients in the hypertension group (1144/1911, 59.9%) were male.

**Figure 1:**
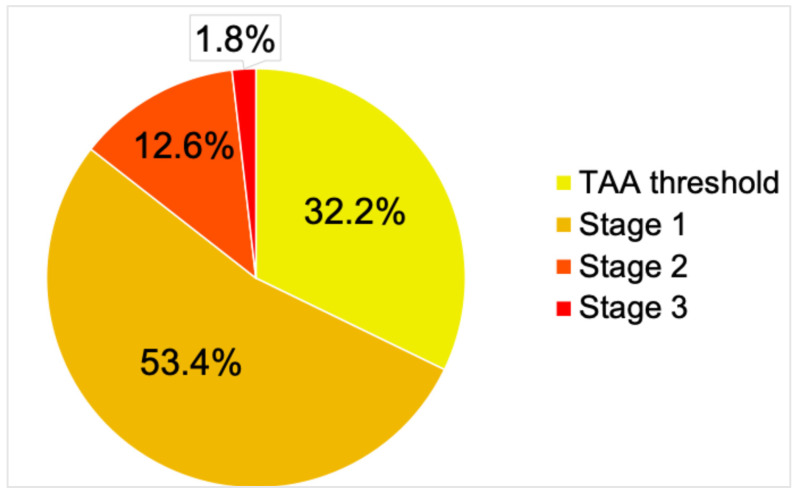
Stages of hypertension. Hypertension stages were defined according to the 2023 European Society of Hypertension (ESH) guidelines. Classifications for hypertension severity begin with the TAA threshold (≥130/80 mmHg), followed by Stage 1 (≥140/90 mmHg), progressing to Stage 2 (≥160/100 mmHg) and Stage 3 (also called “hypertensive crisis”, ≥180/110 mmHg). TAA = thoracic aortic aneurysm.

Among the 3525 patients evaluated, 1911 (54.2%) had above-target BP for TAA. Percentages for the different hypertension stages are depicted in Figure 1.

**Figure 2:**
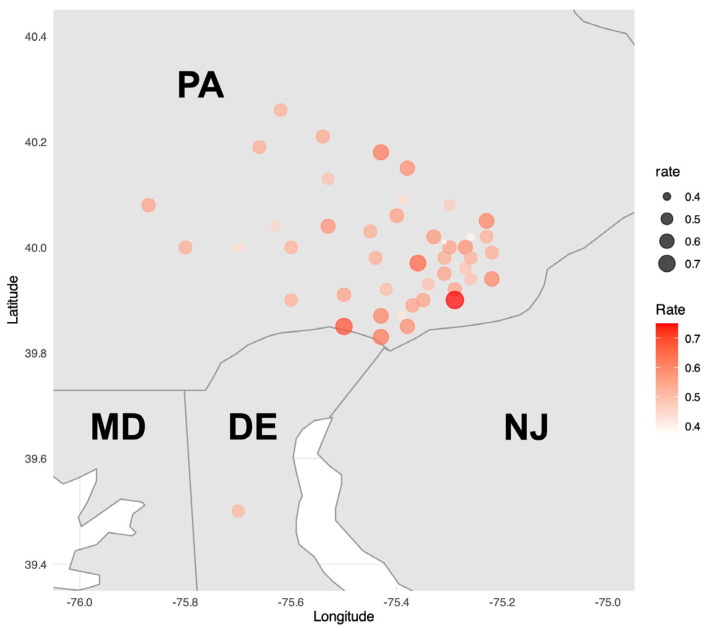
Heatmap of zip codes with at least 20 patients (n = 2536, 46 zip codes). A heatmap illustrating the rate of uncontrolled hypertension by zip code was constructed to visualize geographic distribution. Only zip codes with at least 20 patients were included in the analysis. DE = Delaware; MD = Maryland; NJ = New Jersey; PA = Pennsylvania.

There were 46 zip codes with at least 20 patients (2536/3525, 71.9%). The geographic distribution of uncontrolled hypertension by zip code is depicted in Figure 2.

The results of the univariable regression analysis are reported in Table 1. A trend toward significance was noted for education level, with patients residing in zip codes with a higher proportion of college graduates more likely to fall in the non-hypertensive group, OR = 0.995, *p* = 0.059. Considering only one variable met the inclusion criterion for multivariable analysis, this analysis was not performed.

## 4. Discussion

In this retrospective study of 3525 patients with previously diagnosed TAA referred to a dedicated aortic tertiary-care center, we found that over half (54.2%) of patients had above-target BP, with most of these hypertensive patients (53.4%) falling into Stage 1 hypertension. Elevated BMI was significantly associated with hypertension (*p* < 0.01), and there was a trend toward increased hypertension among patients living in zip codes with lower rates of college graduation (*p* = 0.059). These findings highlight a critical gap in the management of a particularly high-risk cardiovascular population, a gap which exists even with prior healthcare visits.

Blood pressure management in TAA patients should be initiated immediately at diagnosis of aortic aneurysm by any clinician [15,19]. As the single most important predictor of TAA complications, guideline-directed BP control is the most important modifiable risk factor that can impact disease progression in this patient population [2,18]. Adequate hypertension control can benefit these patients in multiple ways, such as slowing aneurysmal growth [6], in addition to reducing life-threatening acute aortic complications and mortality [20]. Indeed, these complications have crucial societal implications, being associated with high costs for the management of aortic dissections and ruptures for the rest of these patient’s lives [21]. Therefore, the international guidelines recommend a treatment target of <130/80 mmHg in patients with aortic disease [9,18]. The recently released 2025 ACC/AHA guidelines further highlight the importance of even more aggressive BP control: while <130/80 mmHg remains the standard, these guidelines now encourage achieving systolic pressures < 120 mmHg when tolerated. They also recommend initiating pharmacologic therapy for patients with systolic 130–139 mmHg if lifestyle measures fail after 3 to 6 months [22]. Therefore, it is even more important to achieve these targets in this particularly vulnerable TAA patient population. Despite this recommendation and a referral to a specialized center, BP was not adequately controlled in more than half of the patients referred for confirmed TAA. This discrepancy raises concerns about the real-world implementation of clinical guidelines, possibly suggesting that patients with high-risk aortic aneurysm may not be receiving adequate BP control at the primary-care level. This inadequacy could stem from poor patient compliance or from an ineffective BP medication regimen, such as incorrect dosage or suboptimal drug selection.

A substantial body of research has explored the various risk factors associated with uncontrolled hypertension in the general population. Demographic and socioeconomic characteristics are crucial factors in influencing hypertension control. Older individuals, particularly those above 65 years, have been found to be at significantly higher risk for uncontrolled hypertension [23]. Sex differences have also been observed, with men generally exhibiting a higher prevalence of uncontrolled hypertension compared to women [24,25]. In line with these findings, three out of five patients in the hypertension group of this analysis were male. Additionally, racial and ethnic disparities exist, as African Americans and Native Hawaiian/Pacific Islanders have been shown to have greater odds of experiencing uncontrolled hypertension when compared to Caucasian individuals [23,26]. Educational level is another important factor. Individuals with lower levels of education are more likely to have difficulty maintaining BP control [24]. A lower educational level may correlate with reduced understanding of disease severity, decreased adherence to medication, or barriers to accessing care. In this regard, our study only revealed a trend: patients living in less-educated areas had a higher likelihood of uncontrolled hypertension. The lack of significance of this parameter may stem from the fact that it is based on zip code-level information, rather than patient-level information. These factors warrant further investigation, and may benefit from targeted community outreach and patient education initiatives.

Lifestyle and behavioral factors contribute substantially to the persistence of uncontrolled hypertension. Dietary habits, particularly failure to adhere to a low-salt diet or the frequent addition of salt to meals, have been identified as significant contributors [25,27], along with as physical inactivity [24], and excessive alcohol consumption [24,28]. Interestingly, current-smoker status has been found to be inversely associated with uncontrolled hypertension, although this relationship is likely influenced by complex interactions with other risk factors and does not negate the broader health risks associated with tobacco use [28].

Clinical and health-system-related variables are closely linked to hypertension control. Major clinical determinants are adherence to antihypertensive medication [29,30]; comorbid conditions, including diabetes mellitus; metabolic syndrome; and atrial fibrillation [29,31]. Access to health care also plays a vital role. In fact, individuals without a regular primary-care provider or health insurance are more likely to have severe uncontrolled hypertension, a pattern particularly evident in minority populations [32]. Longer delays before clinical evaluation at healthcare facilities have also been previously related to poorer BP control [33]. Moreover, greater fluctuations in BP and elevated arterial stiffness emerged as key contributors to target organ damage, suggesting a mutual influence that could hasten the progression of the disease [34]. Improper cuff sizing, particularly in patients with larger arm circumferences, is a major source of BP measurement inaccuracy, with standard cuffs producing the largest systematic errors [35]. Additional procedural factors, such as rapid cuff deflation and incorrect arm positioning, further compromise measurement reliability. Fully automated oscillometric office systems allow repeated BP measurements without staff presence, thereby minimizing observer-related bias [36,37,38]. In the present study, guideline standards were applied when measuring BP. Measurements were taken by trained personnel using an appropriately sized cuff and a calibrated automated sphygmomanometer.

Finally, biological and genetic factors contribute to the risk profile for uncontrolled hypertension; these include including elevated BMI, reflecting overweight and obesity [24,27]. Our analysis identified BMI as a predictor of hypertension in this TAA group. These findings highlight the need for integrating weight management strategies into the care of TAA patients with hypertension, in addition to pharmacological interventions. In addition, certain biological markers, such as elevated ferritin levels and the presence of albuminuria, have been positively associated with uncontrolled hypertension [28].

Recent investigations have begun to shed light on the relationship between uncontrolled hypertension and TAA, an area that has received comparatively less attention in the medical literature. Much of the existing research on hypertension in the context of aneurysmal disease has historically centered on abdominal aneurysms or on connective tissue disorders such as Marfan syndrome. Nonetheless, one mechanistic review has delved into how hypertension may influence specifically the thoracic aorta. This review identified several biological mechanisms, including dysfunction of vascular smooth muscle cells, degradation of the extracellular matrix, oxidative stress, and abnormal cellular signaling, that could drive aneurysm growth in hypertensive patients [39]. A recent meta-analysis confirmed that hypertension is a dominant modifiable risk factor for both the formation and progression of aortic aneurysms, including those affecting the thoracic segment [40]. A large UK Biobank analysis of 495,542 men and women aged 37–73 years over a mean follow-up of 12.3 years identified 3346 incident aortic aneurysm cases [14]. Hypertension was associated with an increased risk of aortic aneurysm overall (HR 1.17, 95% CI 1.08–1.27), and for thoracic (HR 1.23, 95% CI 1.04–1.46) and abdominal (HR 1.16, 95% CI 1.04–1.30) subtypes. Indeed, hypertension is associated with larger aneurysms and faster growth even in patients without a formal hypertension diagnosis [6].

Despite the absence of randomized controlled trials specifically focused on optimal BP targets for individuals with TAA, expert recommendations and guidelines consistently advise maintaining systolic BP below 130 mmHg [2,18,41]. Even stricter control, with targets of systolic pressure below 120 mmHg, may be appropriate if well tolerated.

### Strengths and Limitations

This retrospective study has several strengths, including a large sample size and the use of validated BP measurements. The inclusion of publicly available zip code-level demographic data adds a socioeconomic context to the clinical findings and highlights the need for stricter and earlier BP intervention at the time of diagnosis of TAA by primary-care, cardiology and emergency-room providers.

However, several limitations need to be acknowledged. Demographic characteristics such as education and income were estimated using zip code-level data rather than individual patient-level information. While these proxies can help identify population-level trends, they may not accurately reflect a person’s actual socioeconomic status or educational attainment. Additionally, the study did not collect data on medication adherence, duration of hypertension, resistant hypertension, exact location of the aneurysm, or treatment regimens, all of which are critical factors. Other important factors, including diet, physical activity, and other medical conditions, were also not captured in the analysis. Differences in BP measurement protocols between referral and tertiary-care centers, together with reliance on single-timepoint assessments, may have led to misclassification and potential overestimation of the diagnosis.

Moreover, because the study was conducted at a single tertiary referral center, the findings may not be generalizable to other clinical settings or geographic populations.

## 5. Conclusions

Hypertension remains both highly prevalent and inadequately controlled in patients with thoracic aortic aneurysm, even within specialized care environments. In this study, higher BMI was found to be significantly associated with poor BP control, and zip codes with a lower level of education showed only a trend toward the same. While high-quality interventional studies are still needed, the current evidence underscores the fact that uncontrolled BP is frequently observed among patients with TAA and is strongly linked to increased aneurysm dimensions, faster growth, and a heightened risk of adverse events. Accordingly, these findings emphasize the need for a more comprehensive approach to risk factor management, one that includes not only pharmacologic therapy but also patient education and weight management, to improve outcomes in this high-risk population. Future prospective studies with comprehensive multivariable adjustment to confirm and clarify the observed associations are warranted.

## Figures and Tables

**Table 1:** Univariable regression analysis by zip code.

Predictor	OR [95% CI]	*p*-Value
Household income	0.999 [0.999; 1.000]	0.384
House value	0.999 [0.999; 1.000]	0.195
White people %	1.000 [0.996; 1.004]	0.914
Education ≥ college	0.995 [0.990; 1.000]	0.059

CI = confidence interval; OR = odds ratio.

## Data Availability

Data supporting the findings of this study are available from the corresponding author upon reasonable request, subject to institutional approval.

## Supplementary Materials

The following supporting information can be downloaded at: https://www.mdpi.com/article/10.3390/healthcare14040515/s1, Table S1: STROBE Checklist.

## Abbreviations

The following abbreviations are used in this manuscript:

BMI	Body Mass Index
BP	Blood Pressure
ESH	European Society of Hypertension
TAA	Thoracic Aortic Aneurysm
