# Supplementary material for: Association with Uncontrolled Hypertension in Thoracic Aortic Aneurysm Patients Referred to a Tertiary-Care Center
Source: Healthcare (Basel). 2026 Feb 18;14(4):515. doi: 10.3390/healthcare14040515 (PMC12940768; doi:10.3390/healthcare14040515)
Supplement: Supplementary file 1 [file healthcare-14-00515-s001.zip › healthcare-4133916-supplementary.pdf]

STROBE (STrengthening the Reporting of OBservational studies in Epidemiology) Checklist for authors of CORR

To be used by authors of all observational clinical studies published in CORR. For this purpose a cohort study (the term used by STROBE) is considered a longitudinal study typically reporting outcomes of treatment in one or more cohorts; a case-control study is one identifying factors in outcomes; a cross-sectional study is one to identify the prevalence of factors or characteristics in a population at a single point in time.

This table is modified from and used with the permission of The STROBE Initiative, www.strobe-statement.org.

STROBE Statement—Checklist of items that should be included in reports of ***case-control studies***

	Item No	Recommendation	Please insert check where included or N/A where not applicable
**Title and abstract**	1	(*a*) Indicate the study's design with a commonly used term in the title or the abstract	Page 1 – Abstract
		(*b*) Provide in the abstract an informative and balanced summary of what was done and what was found	Abstract
**Introduction**			
Background/rationale	2	Explain the scientific background and rationale for the investigation being reported	Page 1
Objectives	3	State specific objectives, including any prespecified hypotheses	Page 2
**Methods**			
Study design	4	Present key elements of study design early in the paper	Page 2
Setting	5	Describe the setting, locations, and relevant dates, including periods of recruitment, treatment, follow-up, and data collection	Page 2
Participants	6	(*a*) *Cohort study*—Give the eligibility criteria, and the sources and methods of selection of participants. Describe methods of follow-up<br>*Case-control study*—Give the eligibility criteria, and the sources and methods of case ascertainment and control selection. Give the rationale for the choice of cases and controls<br>*Cross-sectional study*—Give the eligibility criteria, and the sources and methods of selection of participants	Page 2
		(*b*) *Cohort study*—For matched studies, give matching criteria and number of treated and untreated<br>*Case-control study*—For matched studies, give matching criteria and the number of controls per case	N/A

Participants	13*	(a) Report numbers of individuals at each stage of study—eg numbers potentially eligible, examined for eligibility, confirmed eligible, included in the study, completing follow-up, and analysed	Page 2
		(b) Give reasons for non-participation at each stage	Page 2
Descriptive data	14*	(a) Give characteristics of study participants (eg demographic, clinical, social) and information on other treatments and potential confounders	Page 3
		(b) Indicate number of participants with missing data for each variable of interest	Page 3
		(c) *Cohort study*—Summarise follow-up time (eg, average and total amount)	N/A
Variables	7	Clearly define all outcomes, treatments, predictors, potential confounders, and effect modifiers. Give diagnostic criteria, if applicable	Page 2-3
Data sources/ measurement	8*	For each variable of interest, give sources of data and details of methods of assessment (measurement). Describe comparability of assessment methods if there is more than one group	Page 2-3
Bias	9	Describe any efforts to address potential sources of bias	Page 2-3
Study size	10	Explain how the study size was arrived at	Page 3
Quantitative variables	11	Explain how quantitative variables were handled in the analyses. If applicable, describe which groupings were chosen and why	Page 2
Statistical methods	12	(*a*) Describe all statistical methods, including those used to control for confounding	Page 3
		(*b*) Describe any methods used to examine subgroups and interactions	Page 3
		(*c*) Explain how missing data were addressed	Page 3
		(*d*) *Cohort study*—If applicable, explain how loss to follow-up was addressed<br>*Case-control study*—If applicable, explain how matching of cases and controls was addressed<br>*Cross-sectional study*—If applicable, describe analytical methods taking account of sampling strategy	N/A
		(*e*) Describe any sensitivity analyses	N/A

*Give information separately for cases and controls.

Note: An Explanation and Elaboration article discusses each checklist item and gives methodological background and published examples of transparent reporting. The STROBE checklist is best used in conjunction with this article (freely available on the Web sites of PLoS Medicine at http://www.plosmedicine.org/, Annals of Internal Medicine at http://www.annals.org/, and Epidemiology at http://www.epidem.com/). Information on the STROBE Initiative is available at http://www.strobe-statement.org.

Modifications to STROBE Guidelines: We added a fourth column for authors to check inclusion. You must include all items in your manuscript unless the information is not applicable. Information on the study cohort (Items 13 and 14 in the STROBE guidelines) should be provided in Patients and Methods, not in Results; we have omitted the portions of the STROBE guidelines related to Results and Discussion (see our guidelines). The STROBE guidelines were developed for epidemiological studies; "exposed" or "exposure" have been modified with the words "treated" or "treatment."
